# Atlanta Medical and Surgical Journal

**Published:** 1871-04

**Authors:** 


### EDITORIAL AND MISCELLANEOUS.

### ATLANTA MEDICAL AND SURGICAL JOURNAL.

In again presenting the ATLANTA MEDICAL AND SURGICAL JOURNAL to the profession, we have a few words to say as to our reasons for doing so. Of two things in this connection we are well satisfied; there never has been in our experience so great a necessity for a medical journal in the section of country through which it would be expected mainly to circulate, and there never has been a time during the same period when the prospect of sustaining its original columns was so good. These considerations alone, would be sufficient to justify the enterprise.

Besides, the Journal is an Institution, the operations of which have only been suspended by adverse circumstances growing out of the war, having been in continued progress from its commencement, in September, 1855, until the same date in 1861, being afterwards revived in 1866, and continued for two years without an adequate support, in consequence of the impoverished and disorganized condition of the profession in the South.

With an improved interest in the profession, from increased profits, and a new zeal in its members, from a better regulated state of society in connection with a revival of legitimate industry, as we get away from the demoralizing influences of the war, (especially from the almost insane desire for money-making by every conceivable irregularity,) we are sufficiently hopeful of the present, and especially of the future, to induce us again to put the Journal afloat, with the promise to use our best exertions to make it worthy of support, and with the intention of making it a permanent publication.

The design is to make it a vehicle for any member of the profession who may desire to communicate with his professional brethren through its pages, but, at the same time, to absolutely secure contributions of value from many of the best medical men of the country.

Our intention is to present, at no distant day, a corps of contributors to the readers of the Journal, which, with a thorough illustration of its pages by electrotypes, wood-cuts, etc., will make it, in a high degree, attractive.

Another point which we desire particularly to press, is, that the Journal is not to be the organ, or representative of any institution or clique, but will be devoted to the interests of the profession at large, from which source it is expected to draw its contributions and its patronage.

It is not, however, to be understood that there is to be no individuality or independence in its course, as it is our firm and determined purpose to be outspoken upon all matters in which the profession are interested, and to make an effort to correct many evils which have operated to the serious detriment of our calling, especially in the State of Georgia, where, we are grieved to say, the profession as a body have, since the war, taken but little interest in contributing to the advancement of medicine as a science.

We are pleased, however, to be able to say that we have good reasons to believe that a new era is about to dawn upon us in a professional way in this city; indeed, we have been forcibly reminded by the deeply interesting meetings of the Atlanta Academy of Medicine of the *ante bellum* days of the old Atlanta Medical Society, which added greatly in its day, not only to the improvement of the profession in this locality, but contributed so largely to the extraordinary harmony, which prevailed among us at that period, to such an extent, in fact, as to have been the constant subject of remark and congratulation.

We then, believe that we are even now begining to realize this new era of good fellowship, as well as the dawn of a bright day in all that goes to make up an honorable profession. The mass of the medical men of this city have determined in the interest of a common brotherhood, and of a benevolent and noble science, to know no individual or selfish interests—to countenance no jealous or envious purposes—to lay aside all mischief-makers and "professional-cacklers," and to press straight forward to the accomplishment of some purpose worthy of men and physicians.

To these ends, then, we desire to be used by the profession, and for these purposes we should labor, and to their accomplishment, can assure our readers we shall be able to bring an able corps of original co-laborators from various parts of the United States, besides furnishing, through its columns, an abstract of every advance which is being made throughout the world in every department of medicine and surgery.

